# Evaluation of care and clinical outcomes after the implementation of an electronic medical record system for type 1 diabetes management in Rwanda

**DOI:** 10.1080/16549716.2025.2457826

**Published:** 2025-02-03

**Authors:** Nathalie Bille, Dirk Lund Christensen, Knut Borch-Johnsen, Crispin Gishoma, Stine Byberg

**Affiliations:** a Department of Digital Health Solutions, World Diabetes Foundation, Department of Digital Health Solutions, Bagsværd, Denmark; b Department of Public Health, Global Health Section, University of Copenhagen, Copenhagen, Denmark; c Department of Clinical Epidemiology, Steno Diabetes Center Copenhagen, Herlev, Denmark; d Rwanda Diabetes Association, Kigali, Rwanda

**Keywords:** Type 1 diabetes, electronic medical record systems, quality of care, clinical outcomes, Rwanda, sub-Saharan Africa

## Abstract

**Background:**

Electronic medical record (EMR) systems are increasingly used to improve disease management. However, the impact on data quality, quality of care and clinical outcomes for type 1 diabetes (T1D) in sub-Saharan Africa (SSA) has not yet been explored.

**Objective:**

The aim was to evaluate the effect of implementing an EMR system on the quality of care and clinical outcomes for T1D individuals in Rwanda.

**Methods:**

The Rwanda Diabetes Association collected data during quarterly district hospital visits. We evaluated the effect of a newly developed and implemented EMR system by assessing differences in clinical attendance and outcomes 2 years before (pre-EMR: February 2020-February 2022) and after (post-EMR: February 2022–February 2024) the deployment of the EMR system.

**Results:**

We found an increase in the number of individuals examined and the number of consultations conducted post-EMR. There was an increase in data completeness on all parameters; however, we also found that more people did not monitor their blood glucose post-EMR. We found a significant increase in clinical attendance, and a reduction in median HbA_1c_ levels from 81.4 mmol/mol pre-EMR to 63.9 mmol/mol post-EMR (*p* < 0.001).

**Conclusion:**

Several quality and clinical indicators improved after the integration of the EMR system in T1D management. To the best of our knowledge, this is the first study evaluating the impact of using an EMR system on the quality of care and clinical outcomes for T1D individuals in an SSA context. The long-term effect and implications are yet to be explored.

## Paper context

- **Main findings:** After the implementation of the locally developed T1 diabetes EMR system, there was a significant increase in clinical attendance, data completeness, reduction in HbA1c levels, and higher screening rates for diabetes-related complications.
- **Added knowledge:** Demonstrates the effectiveness of the T1 diabetes EMR system in improving clinical attendance, data quality, and diabetes management while identifying areas needing further improvement, such as systolic BP management and glucometer access.
- **Global health impact for policy and action:** Informs policymakers on the benefits of EMR systems for enhancing diabetes care, supports patient monitoring, and helps identify treatment and care gaps to improve health outcomes.

## Background

The global prevalence of type 1 diabetes (T1D) is increasing [1], significantly impacting healthcare systems, particularly in low- and middle-income countries (LMICs) like Rwanda [2,3]. Effective T1D management requires a comprehensive approach including regular blood glucose monitoring, appropriate insulin administration, patient education and continuous monitoring of short- and long-term complications. In sub-Saharan Africa (SSA), loss to follow-up and clinic-visit adherence is a major challenge [2].

The adoption of electronic medical record (EMR) systems has been perceived as a transformative development in healthcare, offering the potential to enhance the quality of care and improve clinical outcomes [4]. EMRs are widely used in high-income countries for systematic collection, storage, and retrieval of patient health information, enabling healthcare professionals (HCPs) to make informed decisions, streamlining workflows, enhancing patient safety and follow-up of treatment [5,6]. Despite these benefits, EMR implementation and their impact on LMICs remain in the early stages and are underexplored, especially for T1D [3,7,8]. In Rwanda, we developed an EMR system with the aim of improving the registration and management of T1D [9]. The EMR system was developed in close collaboration between HCPs from RDA, a software development team and experts within the T1D care pathway. The system both serves as a T1D registry, storing all patient data, and functions as a decision-support tool for HCPs to improve patient care and clinical outcomes [9]. The system includes key features such as user authentication, patient appointment management, Short Message Service (SMS) notifications and reminders, clinical data validation features, and management prompts, as well as the generation of summary reports at both aggregated and individual level [10]. A detailed description of the EMR system, its features and a logic model about the system assumptions can be found elsewhere [9]. We previously conducted an implementation evaluation, which indicated that the system was preferable over paper forms in improving data collection and work procedures. HCPs expressed satisfaction with using the system and believed it improved data quality and care for individuals with T1D [10]. In this study, we evaluated the effect of the EMR system on the quality of care and clinical outcomes for T1D individuals in Rwanda.

## Methods

### Study population

We included individuals with T1D in Rwanda examined by the RDA at their clinic in Kigali or one of the 41 district hospitals. Individuals with symptoms of T1D are typically diagnosed at district hospitals or the Rwanda Diabetes Association (RDA) clinic, or after referral from local health centers [11–13]. Diagnostic confirmation was based on HbA_1c_ level of ≥48 mmol/mol, random plasma glucose level ≥11.1 mmol/L or fasting plasma glucose ≥7.0 mmol/L, along with information about age at diagnosis, insulin response, symptoms of diabetic ketoacidosis or underweight (<18.5 kg/m^2^). C-peptide and autoantibody testing were not available [11].

### Data collection

RDA is responsible for continuous follow-up on individuals with T1D. In collaboration with the district hospitals, RDA organizes quarterly and annual visits to the hospitals to do clinical examinations, provide essential supplies, such as insulin and glucose test-strips, diabetes care and education, and free of charge. T1D individuals also have access to basic monthly care provided by the NCD nurse at the district hospital. Since 2009, RDA has collected and stored T1D data from quarterly and annual clinical examinations on paper forms, later then entered into an Excel database [13]. Post-EMR deployment in 2022, all data were recorded directly in the system [9]. However, district hospitals are not using the EMR system, so data from these visits continue to be recorded separately. Individuals treated at the district hospitals who do not attend the RDA’s visit may not be included in the RDA’s EMR.

### Study design

We evaluated the effect of the EMR system by comparing the clinical attendance pre-EMR (February 2020-February 2022) and post-EMR (February 2022-February 2024). We included values during a two-year period before and after EMR implementation, as not all individuals attend clinical consultations regularly (every third month). Pre-EMR measurement was defined as the last measurement conducted pre-EMR, but within 2 years before the EMR deployment, to give the most recent status of the patient before the implementation. Post-EMR measurement was defined as the last measurement within 2 years after the implementation (February 2024). We included the last measurement to ensure the most recent data for everyone and to ensure that there was sufficient time to show the potential effects of EMR. Both data collection procedures and registries have been detailed elsewhere [9,11].

### Data definition

We assessed the differences in clinical and quality-of-care indicators before and after the EMR implementation. Clinical indicators included BMI, HbA_1c_, systolic and diastolic blood pressure (BP). Height was measured without shoes using an SECA stadiometer (Fazzini, Vimodrone (MI), Italy) by a nurse. Weight was measured with an SECA non-electronic floor scale with clothes but without shoes. HbA_1c_ was measured by the Siemens Healthineers DCA vantage® analyzer (Tarrytown, NY, USA). The machine reports the results within the range from 2.5% to ≤14.0%. Values above 14.0% are reported as >14.0%. The values were converted from % (NGSP) to mmol/mol (IFCC) to follow the International Consensus Statement of US and European Diabetes Associations´ recommendation [14]. The treatment target was defined as HbA_1c_ ≤64 mmol/mol (RDA Director, Rwanda National Institute of Health) [15]. Systolic and diastolic BP was measured on left upper arm with ‘Omron M2 basic’ automatic BP monitor (Omron Healthcare Co. Ltd., 24, Yamanouchi Yamanoshita-cho, Ukyo-ku, Kyoto, 615–0084, Japan). A second measurement was taken if values were above the treatment targets defined as: systolic BP ≤120 mmHg and diastolic BP ≤80 mmHg. The last measurements were registered in the EMR system.

Quality-of-care indicators included yearly clinic visits, self-reported daily glucose monitoring, self-reported daily insulin injections and screening for complications (nephropathy and neuropathy). Screening for retinopathy and diabetes-related distress was not conducted in the pre-EMR system and was not included as an evaluation parameter. Nephropathy screening was assessed as the presence of an albumin–creatinine ratio (ACR) test, serum creatinine or proteinuria test (no/yes). Neuropathy screening was conducted using monofilament or tuning fork.

### Statistical analysis

**Figure 1.**
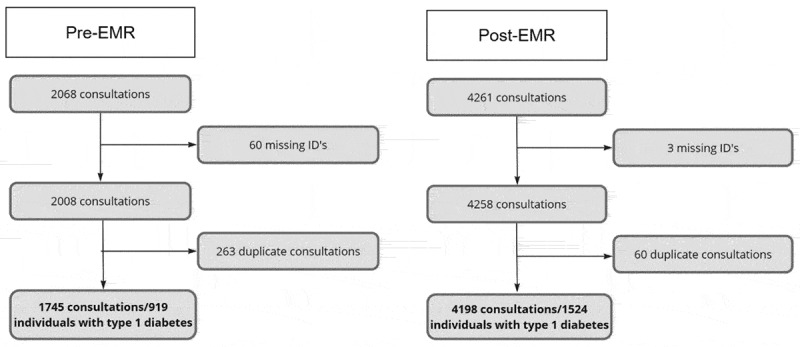
Data exclusion flow chart pre-EMR and post-EMR.

Analyses were performed using R-studio. Duplicate registrations and registrations without an ID were excluded. The total number of consultations conducted pre- and post-EMR implementation was calculated (Exclusion flowchart, Figure 1). Data were checked for missing values before and after EMR-implementation. Descriptive statistics were calculated for all variables. Frequencies were calculated for categorical variables and mean (SD) for normally distributed variables and median (IQR) for non-normally distributed variables. We compared clinical and quality indicators before and after EMR implementation using χ2-test to assess differences among categorical variables and two-sample *t*-test (normal distribution) and Wilcoxon tests (non-normal distribution) to assess differences in continuous variables. P-values <0.05 are used to denote statistical significance.

## Results

### Data inclusion

The number of individuals included for analysis is shown in the flowchart in Figure 1. It shows the total number of consultations 2-year pre- and post-EMR implementation.

Pre-EMR, RDA conducted 1745 consultations, and 919 T1D individuals were examined over a two-year period. Post-EMR, 4198 consultations were conducted, and 1524 individuals examined over a consecutive two-year period, corresponding to a 66% increase from pre- to post-EMR. The post-EMR data had fewer missing IDs and fewer duplicate registrations of consultations, indicating improved data quality.

Table 1 shows the distribution of demographic characteristics pre- and post-EMR. The proportion of males increased from 45.8% to 47.1%, and individuals from Kigali province increased from 17.8% to 21.3%, while the Southern region generally had a higher number of T1D individuals compared to the other regions. We found a difference in mean age of 2.2 years between pre-EMR and post-EMR, corresponding to the two-year difference in data-collection (2020–22 vs 2022–2024). No significant difference in height, weight and BMI from pre- to post-EMR was found. In both periods, the median BMI was at the lower level (BMI: 21 kg/m^2^) of normal range.

**Table 1.** Study population – demographic characteristics.

	Pre-EMR	Post-EMR	P-value
	Total = 919 Individuals (100%)	Total = 1524 Individuals (100%)	
N consultations	N = 1722	N = 4198	
Sex N (%)			
Male	421 (45.8%)	718 (47.1%)	*p* = 0.03*
Female	494 (53.8%)	806 (52.9%)	
Missing	4 (0.4%)	0 (0.0%)	
Province N (%)			
Kigali	164 (17.8%)	324 (21.3%)	*p*<0.001*
North	145 (15.8%)	197 (12.9%)	
South	269 (29.3%)	414 (27.2%)	
East	145 (15.8%)	346 (22.7%)	
West	164 (17.8%)	233 (15.3%)	
Missing	32 (3.5%)	10 (0.7%)	
Age (years) Mean (SD)	20.3 (5.54)	22.5 (6.7)	P<0.001*
Height (cm) Median (IQR)	157 (150–165)	159 (151–165)	*p* = 0.06
Weight (kg) Median (IQR)	53 (44–60)	54 (46–61)	*p* = 0.05
BMI Median (IQR)	21 (19–23)	21 (19–23)	*p* = 0.57

P-values of < 0.05 was used to denote significance*.

Table 2 shows the distribution of the quality-of-care indicators and clinical outcomes pre- and post-EMR, showing improved data completeness from pre- to post-EMR for all quality-of-care indicators. We found a higher clinical attendance from one median yearly visit pre-EMR to 1.5 post-EMR.

**Table 2.** Population characteristics on quality-of-care indicators and clinical outcomes for individuals with T1D pre- and post-EMR.

	Pre-EMR	Post-EMR	P-value
	Total = 919 Individuals	Total = 1524 Individuals	
N consultations	N = 1722	N = 4198	
**Quality of care indicators**			
Glucose monitoring (N%)			
No	48 (5.2%)	651 (42.7%)	*p*<0.001*
Yes	643 (70.0%)	826 (54.1%)	
Missing	228 (24.8%)	47 (3.1%)	
Daily glucose monitoring (Mean (SD))	2.2 (0.8)	2.3 (1.3)	*p* = 0.19
Daily insulin injections			
0	6 (0.7%)	23 (1.5%)	*p*<0.001*
1	37 (4.0%)	63 (4.1%)	
2	688 (74.9%)	1028 (67.5%)	
≥3	181 (19.7%)	410 (26.9%)	
Missing	7 (0.8%)	0 (0%)	
Daily insulin injections Mean (SD)	2.1 (0.5)	2.2 (0.7)	*p* = 0.10
Clinic visits			
1	414 (45.0%)	433 (28.4%)	*p*<0.001*
2	289 (29.3%)	315 (20.7%)	
3	161 (17.5%)	285 (18.7%)	
4	67 (7.3%)	243 (15.9%)	
5	6 (0.7%)	187 (12.3%)	
≥6	2 (0.2%)	61 (4.0%)	
Missing	NA	NA	
Yearly clinic visits Median (IQR)	1 (0.5–1.5)	1.5 (0.5–2)	*p*<0.001*
Nephropathy screening			
No	524 (57.2.%)	1076 (70.6%)	*p*<0.001*
Yes	101 (10.9%)	442 (29.0%)	
Missing	294 (31.9%)	6 (0.4%)	
Neuropathy screening			
No	401 (43.6%)	717 (47.0%)	*p* = 0.6
Yes	461 (50.2%)	785 (51.5%)	
Missing	57 (6.2%)	22 (1.5%)	
**Clinical parameters**			
SBP (mmHg) Mean (SD)	112.4 (15.7)	114.0 (16.1)	*p* = 0.02*
DBP (mmHg)	71.8 (11.0)	72.1 (10.8)	*p* = 0.42
HbA1c (%) Median (IQR)	81.4 (62.8–107.7)	63.9 (55.2–85.8)	*p*<0.001*

P-values of < 0.05 was used to denote significance*.

Pre-EMR, 24.8% had missing information on blood glucose monitoring, whereas this was 3.1% post-EMR. There was a higher share of individuals who did not monitor their blood glucose levels post-EMR (42.7%) vs pre-EMR (5.2%). For daily insulin injections, we found a slightly higher proportion with ≥3 daily insulin injections post-EMR compared with pre-EMR (19.7% vs. 26.9%). There was no significant difference between pre- and post-EMR mean daily insulin injection (2.1 vs 2.2) and mean daily glucose monitoring (2.2 vs 2.3), respectively. Fewer missing data were seen post-EMR compared to pre-EMR for complications screening. Especially for nephropathy, missing data decreased from 31.9% pre-EMR to 0.4% post-EMR and nephropathy screening also increased from 10.9% of patients pre-EMR to 29.0% post-EMR. In the clinical indicators, we found no difference in diastolic BP but a slightly higher systolic BP from pre- to post-EMR. HbA_1c_ levels decreased significantly from 81.4 mmol/mol pre-EMR to 63.9 mmol/mol post-EMR (p < 0.001**).

Table 3 shows that a higher proportion of the T1D population was within target (≤64 mmol/mol) of glycemic control post-EMR (52.6%) compared to pre-EMR (28.0%). Post-EMR, we observed a notable decrease in the proportion of T1 individuals achieving the targeted systolic BP. No significant change was observed in the diastolic BP.

**Table 3.** Population within the target of key clinical indicators.

	Pre-EMR	Post-EMR	χ^2^ -test p-value
N Individuals	N = 919	N = 1524	
HbA_1c_ (≤64mmol/mol)			
Yes	258 (28.0%)	801 (52.6%)	*p*<0.001**
No	654 (71.2%)	720 (47.2%)	
Missing	7 (0.8%)	3 (0.2%)	
Systolic BP (≤120 mmHg)			
Yes	648 (70.5%)	857 (56.2%)	*p*<0.001**
No	262 (28.5%)	667 (43.8%)	
Missing	9 (1%)	0 (0.0%)	
Diastolic BP (≤80 mmHg)			
Yes	724 (78.8%)	1 258 (82.5%)	*p* = 0.1*
No	185 (20.1%)	266 (17.5%)	
Missing	10 (1%)	0 (0.0%)	

P-values of < 0.05 was used to denote significance*.

## Discussion

After the implementation of the EMR system, we observed an increase in individuals examined, consultations per patient with T1D, as well as improved data completeness. We also found no difference in the median daily number of glucose monitorings from pre- to post-EMR but noted that more T1D individuals did not monitor their glucose levels post-EMR. More individuals were screened for diabetes-related complications post-EMR, and data completeness increased on screening status for all complications. We found significantly lower median HbA_1c_ levels post-EMR; however, we did see higher levels of systolic BP from pre- to post-EMR.

### T1D records and data completeness

The higher number of individuals with T1D and clinical consultations after the implementation of EMR system could be attributed to either an increase in diagnosed individuals and consultations or a reflection of an actual increase in T1D prevalence in Rwanda. According to the IDF Diabetes Atlas 2021, the number of T1D individuals (age <20) in Africa has more than doubled since 2019, likely due to improved diagnosis and availability of new data [16]. Studies from Tanzania also showed an increase in incidence and total numbers of T1D individuals, from approximately 1 000 in 2006–2010 to more than 4000 in 2022 [17–19]. The authors suggested that the increase was a combined result of improved awareness, diagnosis and expansion of T1D care into primary care facilities [19]. These factors might also have contributed to the increased numbers in Rwanda. Moreover, the increase in the number of individuals and consultations can also be attributed to better record-keeping and data registration enabled by the EMR system. Pre-EMR, data were recorded on paper forms and later entered into an Excel database, which could lead to incomplete or missing records. The EMR system allows for direct data entry, improving accuracy and completeness and likely contributing to higher numbers of observed post-EMR.

Improved coordination of care between district hospitals and RDA facilitated by the EMR system has likely helped identify and register T1D individuals who might have previously gone ‘under the radar.’ This improved coordination ensures that these individuals receive appropriate follow-up and care, further contributing to the increase in diagnosed T1D individuals. The average age of the post-EMR group being 2.2 years older than the pre-EMR group suggests that the EMR system may have helped identify older individuals who were previously undiagnosed or unregistered. This age difference, coupled with an average two-year gap between pre- and post-EMR implementation, implies that the system’s implementation has played a significant role in capturing a more comprehensive patient population. Moreover, the EMR system improving care and follow-up may have led to improved survival and consequently an increase in prevalence. Therefore, the increase in prevalence could reflect both improved detection and improved follow-up and survival, rather than an actual increase in T1D incidence.

Simultaneously with the EMR system roll-out, RDA conducted awareness activities throughout the country. Additionally, the Rwanda Biomedical Centre (the implementing partner of the Ministry of Health) has increased efforts to expand T1D care to the primary care level. Despite these efforts, RDA reports that the number of HCPs and employees has not increased, suggesting that the rise in consultations is due to improvements in data registration, efficiency, and accuracy of T1D management and follow-up.

The literature is inconclusive on whether EMR system improves efficiency [20]. A study from the US found no difference in time spent on patient-related tasks between nurses using and not using an EMR system [21]. Another study, evaluating the EMR system on the use effect on nursing activities, reported a 30% reduction in documentation time (20.5% using paper forms vs 14.4% using EMR systems), corresponding to 29 min per 8 h nursing shift [22]. A review including 16 studies from SSA found that efficiency in EMR systems was associated with reduction of delays, burden of waiting, and improvements in workflows [8]. None of the 16 systems reviewed received an excellent rating for efficiency, 4 received a good rating, 11 a fair rating, and 1 a poor rating. Moreover, most of the systems reviewed were found to effectively achieve implementation objectives, including improving data quality and records availability [8,23].

We observed more consultations and improved data completeness post-EMR, likely due to a structured and standardized data capture process, reducing errors and inconsistencies common with paper records [24]. Moreover, the use of the EMR system may save time by reducing manual and repetitive data entry, allowing for more consultations. The EMR system had built-in mandatory fields and prompts that helped ensure that all necessary data was collected accurately. A study from Kenya reported significant improvements in data capture and completeness with EMR systems, reducing errors by flagging incorrect values [24].

### Quality-of-care

Post-EMR, we found that more individuals never monitored their blood glucose compared to pre-EMR. This may be due to better registration of individuals without access to glucometers or that fewer patients have a working glucometer post EMR and therefore have no measurements. Nevertheless, the EMR helped identify that a large percentage (42.7%) of the study population had no daily glucose monitoring – information that was not available pre-EMR and can guide treatment improvements and national policy.

T1D individuals in Rwanda are recommended to attend monthly visits at the district hospitals and quarterly visits with the RDA [11]. This study found that the frequency of clinic visits with RDA increased from a median of once-a-year pre-EMR to 1.5 post-EMR. The percentage of individuals who had ≥4 visits increased from 8.2% pre-EMR to 32.2 post-EMR. The increase in attendance might be attributed to improved coordinated care, facilitated by the EMR system’s structured patient overview, including their contact information. Additionally, the system’s capability to send SMS reminders about upcoming consultations and alert HCPs of patients lost-to-follow-up likely contributed to this increase. Despite improvements, quarterly attendance targets are not met. Studies from SSA within other disease areas showed similar results [25]. One study concluded that retention and follow-up of individuals with HIV in antiretroviral therapy are still unsatisfactory, even though improved with the use of EMR system. Other studies showed that more than 20% of individuals with HIV had missed appointments or were lost-to-follow-up in Kenya after 1 year [26], up to 59% in Malawi after 4 years [27], and 80% of babies born to HIV-positive mothers were lost-to-follow-up after 1 year in South Africa [28]. It has been suggested that follow-up was best achieved with a combination of EMR system reports and collaboration with community health workers [26]. Although EMR systems help track and improve clinical attendance, they cannot alone solve the issue of loss-to-follow-up.

In addition to an increase in clinic visits, we also found a post-EMR improvement in data availability and screening status of diabetes-related complications. Nephropathy screening increased from 10.9% pre-EMR to 29.0% post-EMR, likely facilitated by the EMR system’s ability to set reminders for HCPs about upcoming screenings. The reminders serve as a prompt for HCPs, reinforcing the importance of regular complications screening and ensuring it is a consistent part of the care plan. In our study, we found a variation in screening status depending on the type of complications. Despite this increase in nephropathy screenings, it was still less frequently conducted than neuropathy screenings. Interestingly, the frequency of neuropathy screenings remained unchanged from pre- to post-EMR. Contributing to its more consistent usage, the screening tool for neuropathy (e.g. monofilament or tuning fork) might have been easier to administer, more accessible and less time-consuming, than for nephropathy screening (ACR measurement requiring a urine test and cartridges).

We found a high level of data completeness for HbA_1c_ testing both pre- and post-EMR, suggesting that HbA_1c_ testing has been continuously available in Rwanda. The lower median HbA_1c_ levels seen post-EMR could be partly reflected by an increase in individuals reporting to have ≥3 insulin injections per day post-EMR (26.9%) compared to pre-EMR (19.7%). The system highlights increases in core values, such as HbA_1c_, alerting HCPs to negative trends and prompting them to adjust treatment plans accordingly. Furthermore, we saw an increase in clinic visits, which could also indicate an improved treatment adherence, leading to reduced HbA_1c_ levels. A study of T1D individuals from Israel (aged 18–21 years) found higher clinical attendance rates (number of clinic visits/number of scheduled visits) significantly associated with better HbA_1c_ control [29]. Other parallel initiatives, such as the Ministry of Health Rwanda’s increased focus on improving non-communicable disease care, including T1D, may have contributed to the HbA_1c_, reduction by improving access to diabetes medicines and diabetes education. In November 2022, RDA in collaboration with the Rwanda NCD Alliance and Youth Voice for Change launched a project to improve T1D care in rural and suburban areas. The initiative includes training volunteers to educate young T1D patients and their families, as well as raising community awareness and advocating for T1D through media engagement [30].

Surprisingly, we found a large increase in individuals with off-target (≤120 mmHg) systolic BP from pre-EMR 28.5% to 43.8% post-EMR. Noting that the target level of ≤120 mmHg might be somewhat conservative in individuals with diabetes, and in some studies have been classified as pre-hypertension [31,32], we adjusted the target level to ≤130 mmHg. The overall result did not change significantly, suggesting that the increase in off-target BP levels is not solely dependent on the specific target threshold used. Additional analyses found that neither age, diabetes duration nor newly diagnosis could explain the higher prevalence of hypertension found post-EMR. BP values have been shown to be sensitive to modifications in the measurement methodology, which could introduce systematic errors [33]. We are unaware of any major changes in BP measurement procedures that could have affected the results. RDA is responsible for measuring patients’ BP, and patients with hypertension are referred to specialized hypertension clinics for treatment. Since the RDA did not prescribe hypertension medication, we lack data on potential changes in medication supply or prescription patterns that could potentially explain an increase in the systolic BP.

### Strengths and limitations

The strength of this study is that by comparing data from 2 years before and after the EMR system, we were able to observe changes occurring after the implementation. Some effects might not be immediately apparent after the implementation but may become evident over time. According to RDA, there have been no significant changes in T1D care delivery in the 2 years before and after implementing the EMR.

A limitation could be that quality-of-care and clinical outcomes can be influenced by many factors, and other initiatives besides the EMR system may have contributed to the observed improvements. Since this was a ‘real-world’ study without a control group, we cannot determine whether the observed effects were due to the EMR system or other overarching factors occurring during the same period. We could have more confidently attributed the improvements in quality of care and clinical outcomes to the EMR system if we had conducted a cluster randomized controlled trial (RCT) or a step-wedge study, where the intervention is rolled out over different periods of time in different clusters [34]. Nevertheless, this was not feasible in the current context, as the roll-out of the EMR system was part of a larger quality-of-care improvement initiative, aiming to improve the quality of diabetes care for T1D in a similar manner for all. All data migration from Excel-file to EMR was done in a few days, and HCPs were trained simultaneously.

The absence of diagnostic confirmation through C-peptide or autoantibody testing, crucial for confirming T1D, presents another limitation. Without these tests, it is possible that some individuals with other phenotypes of diabetes (e.g. MODY or MRDM) could be misclassified as having T1D in the EMR system. Nonetheless, given that the average age and BMI in this group was low and that they all need insulin treatment, we expect that the presence of other diabetes types is limited. Unfortunately, we lack data regarding insulin types and any changes during the study. The potential replacement of older insulin types with newer, more modern options could have also played a role in the observed reduction in HbA_1c_. Furthermore, it seemed as if there were some country-level differences in who was entering the study and regional differences in the number of identified T1D individuals. However, we did not observe any regional variation in the effect of HbA_1c_ level pre- and post-EMR.

Another limitation was the potential systematic bias from the HbA_1c_ tool’s inability to measure above 14%. This could lead to an underestimation of the median HbA_1c_ level and diabetes severity in the population, as well as an underestimation of the true difference in HbA_1c_ levels pre- and post-EMR.

## Conclusion

After implementing the T1D EMR-system in Rwanda, we observed an improved data quality, higher frequency of clinic visits, and higher screening frequencies for diabetes-related complications. Furthermore, we observed that the HbA_1c_ levels were lower after the EMR implementation. The EMR system proved to be a useful tool in monitoring the progression of the disease, its related complications and identifying treatment gaps. For example, we identified more individuals not monitoring daily glucose levels and an increase in the frequency of off-target systolic BP. The continued use of the EMR system can contribute to increased knowledge about T1 epidemiology, including incidences, prevalence, risk factors and optimal treatment, which are still relatively unknown in much of the SSA region. This information can guide policymakers in creating evidence-based health policies, identify health disparities and inform resource allocation.
